# A retrospective analysis of factors influencing response to omalizumab treatment in Indian patients with antihistamine refractory chronic spontaneous urticaria

**DOI:** 10.5415/apallergy.0000000000000184

**Published:** 2025-02-10

**Authors:** Thammannagowda Prarthana, Hitaishi Mehta, Anuradha Bishnoi, Davinder Parsad, Muthu Sendhil Kumaran

**Affiliations:** 1 Department of Dermatology, Venereology, and Leprology, Postgraduate Institute of Medical Education and Research, Chandigarh, India

**Keywords:** 7-day urticaria activity score, antihistamines, chronic refractory urticaria, chronic spontaneous urticaria, omalizumab, serum IgE level, wheals

## Abstract

**Background:**

Chronic spontaneous urticaria (CSU) presents as a persistent and distressing condition, with varying treatment responses. Omalizumab, a monoclonal anti-IgE antibody, has shown efficacy in managing antihistamine (AH_1_)-refractory CSU, but its varied response patterns and associated factors remain understudied, particularly in India.

**Methods:**

We conducted a retrospective study involving 81 antihistamine-resistant CSU patients treated with omalizumab at a tertiary care center in Northern India between 2018 and 2023. Baseline characteristics, treatment response, and adverse effects were analyzed. Patients were categorized into various response groups based on treatment timelines and biomarker correlations.

**Results:**

We observed 65% achieved symptom cessation (group 1) following a single omalizumab dose, while 21% responded between second and third doses (group 2). A subset (7.4%) necessitated increased dosing frequency (group 3) for symptom control. Additionally, 6.2% showed persistent symptoms despite increased dosing frequency (group 4), exhibiting distinctive biomarker profiles indicative of an autoimmune endotype. Notably, 27.1% experienced exacerbations during treatment, emphasizing the need for tailored management approaches and response expectations.

**Conclusion:**

Omalizumab demonstrated remarkable efficacy in the treatment of AH_1_-refractory CSU, with a good safety profile. This study highlights the complexity of treatment response to omalizumab and the potential utility of biomarkers in guiding personalized therapeutic strategies. Further research into biomarker-based endotypes is warranted to optimize CSU management.

## 1. Introduction

Urticaria is an autoimmune disorder characterized by wheals and itching with or without concomitant angioedema inflicting a significant psychosocial burden on patients and their families. Chronic spontaneous urticaria (CSU) is defined as the occurrence of wheals, angioedema, or both for more than 6 weeks, in the absence of any definitive triggers [1]. The first-line symptomatic treatment option for CSU is second-generation H1-antihistamines (AH_1_) in standard doses. However, only 50% respond to first-line treatment and may require dose escalation of AH_1_. A subset of patients remains refractive even after updosing [2, 3]. The international European Academy of Allergology and Clinical Immunology (EAACI), the EU-founded network of excellence, the Global Allergy and Asthma European Network, the European Dermatology Forum and the World Allergy Organization urticaria guideline recommends the addition of omalizumab as a second-line therapy in patients who are refractory to AH_1_ at full dose [4]. Omalizumab is a monoclonal anti-IgE antibody that binds to free IgE, which lowers free IgE levels and subsequent downregulation of FcεRI on basophils and mast cells. The response to omalizumab exhibits variability across different subsets of patients with CSU. Patients can be classified as either early or late responders, determined by the timing of their response to omalizumab. Moreover, response categorization involves further distinctions, like labeling patients as complete, good, or partial responders, which is based on the degree of reduction observed in the 7-day urticaria activity score (UAS7). Nonresponders are also identified within this framework [5, 6]. Notably, individuals with elevated IgE levels demonstrate a prompt response, whereas those presenting with features associated with autoimmunity exhibit a comparatively gradual response to treatment [7].

While real-world studies on omalizumab are primarily conducted in Western countries, there exists a significant lack of data from the Indian subcontinent. This retrospective chart review aims to address this gap by delineating the treatment response patterns to omalizumab in patients with CSU in India. Additionally, the study seeks to establish correlations between clinicodemographic profiles of patients and their responses to omalizumab within the context of a real-world setting.

## 2. Methodology

### 2.1. Study setting

We conducted a retrospective study in patients with CSU treated with omalizumab between January 2018 and December 2023 at a tertiary UCare center that caters to urticaria patients from 5 northern states of India. The data were retrieved retrospectively from patients’ medical records in the urticaria clinic. As a part of our standard protocol, all patients diagnosed with CSU undergo baseline assessments, including levels of serum IgE, vitamin D, d-dimer, C-reactive protein (CRP), antithyroid peroxidase (TPO) antibodies, and antinuclear antibodies (ANA). We follow the recommendations outlined by the EAACI for the management of urticaria [1]. Patients are started on standard dose of AH_1_, with dose escalation in nonresponders. In patients not responding to 4-fold increase in AH_1_ dosages, injection of omalizumab at a dose of 300 mg is administered subcutaneously every 28 days. Follow-up visits every 4 weeks include a thorough assessment of UAS7, urticaria control test (UCT) scores, and vigilant monitoring for any adverse effects. Following the administration of 3 doses of omalizumab, if the urticaria is effectively managed, the same standard dosing schedule was continued. However, if the symptoms persist, we modified the frequency of administration to every 21 days.

### 2.2. Ethics

We conducted a retrospective study involving the analysis of anonymized patient data. Given the nature of our research, we sought approval from the ethics committee at our institution for a waiver of consent. This waiver allowed us to access and utilize the data while maintaining patient privacy and adhering to ethical guidelines.

### 2.3. Study population

In the absence of a universally accepted definition for chronic refractory urticaria (CRU), we defined CRU as CSU persisting for at least 6 months [3], along with lack of control despite the administration of a fourfold increased dosage of AH_1_ for at least three consecutive months, thereby requiring repeated short-term courses of oral corticosteroids or other immunomodulators [8].

All patients >18 years of age, diagnosed with CRU, who completed at least 6 doses of omalizumab were included in the analysis. This is because the EAACI guidelines recommend evaluation of response to omalizumab after at least 4 to 6 months of treatment [9]. Exclusion criteria included patients with incomplete or insufficient medical records hindering a comprehensive retrospective analysis.

### 2.4. Data collection

Data collected included baseline characteristics, incorporating age, gender, duration, and type of disease. Baseline laboratory assessments, including serum IgE, vitamin D levels, d-dimer, CRP, antithyroid peroxidase (anti-TPO) antibodies, and antinuclear antibodies (ANA), were recorded in a predefined proforma. UAS7 and UCT scores of each follow-up visit during the study duration were recorded. UAS7 scores were defined as: urticaria-free (0); well controlled (1–6); mild (7–15); moderate (16–27); and severe (28–42). Change in UAS7 after starting omalizumab was determined [10]. Additionally, documentation included the dosage of omalizumab administered, the frequency of its administration, and any adverse effects observed throughout the treatment period.

### 2.5. Assessment of treatment response

In accordance with the consensus of a group of experts, treatment response was characterized by achieving UAS7 ≤ 6 [6], and the time to response was recorded for each patient. Patients were categorized based on the timing of treatment response:

(1) Group 1: early responders showing improvement after the first dose within 4 weeks.
(2) Group 2: late responders exhibiting a response after 4 weeks but before 12 weeks.
(3) Group 3: responders to frequent dosing achieving improvement after 12 weeks but before 24 weeks, following a reduced dosage interval to 3 weeks.
(4) Group 4: nonresponders did not show improvement even after increasing dosage frequency up to 24 weeks.

Moreover, occurrences of relapses, if any, following the initial treatment response were documented throughout the 24-week study period.

### 2.6. Statistical analysis

Data were analyzed using a statistical package for the social sciences (SPSS, IBM, Chicago, Illinois, USA) (Version 26.0). Normal distribution of continuous data was checked by the Shapiro–Wilk test. Mean and standard deviation (SD) were employed for normally distributed continuous variables. Median and interquartile range (IQR) were calculated for skewed data. Frequencies and percentages were calculated for categorical data. Kruskal–Wallis test and chi-square test were used to assess the relation between baseline parameters and response to omalizumab treatment for quantitative and categorical variables, respectively. A significance level of *P* < 0.05 was chosen to determine the statistical significance of the observed associations.

## 3. Results

**Figure 1.**
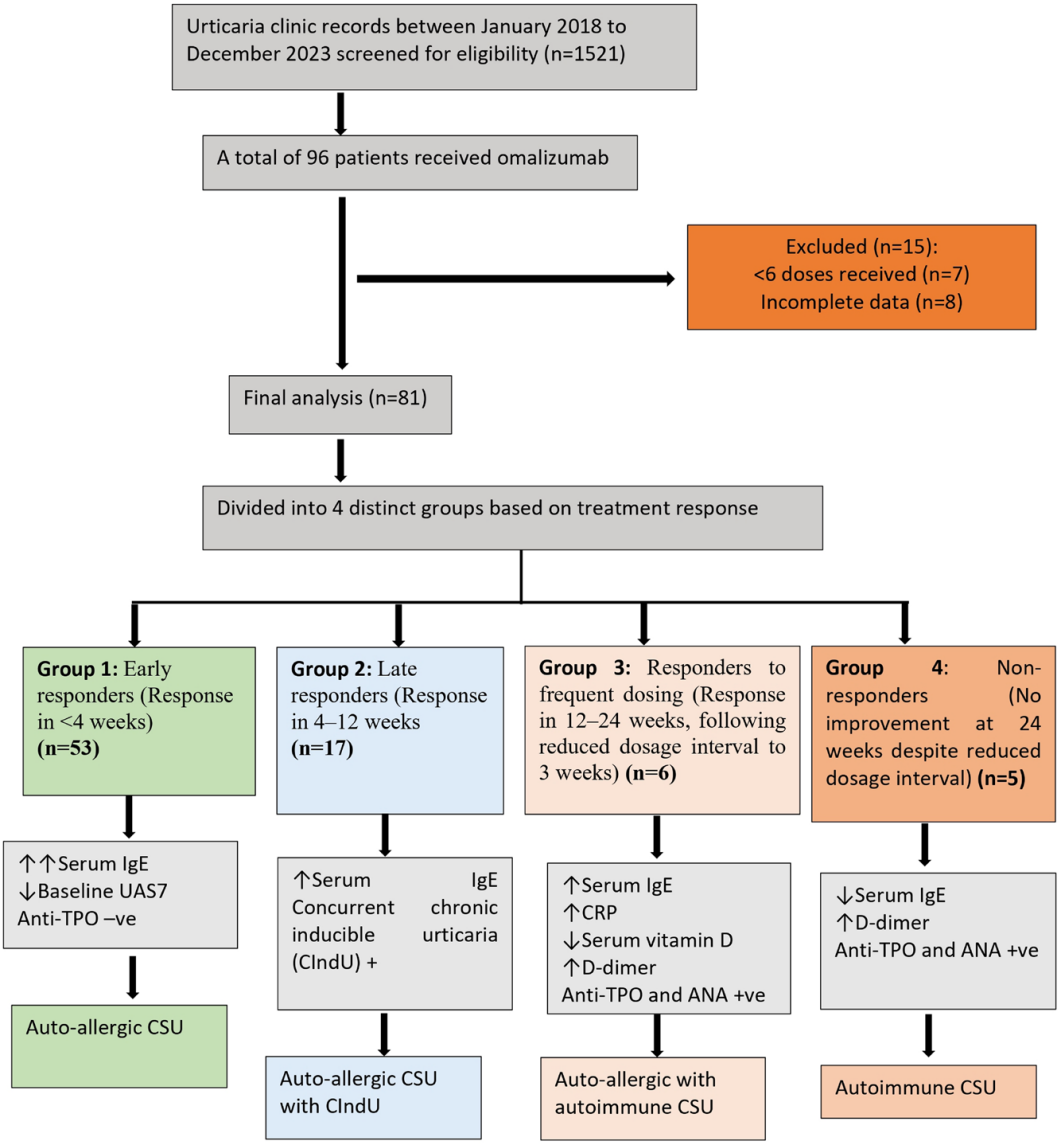
Flow chart depicting groups of study patients.

A comprehensive screening of 1,521 records yielded 96 patients who had received omalizumab. Among these, after applying the comprehensive inclusion and exclusion criteria, 81 patients who had received a minimum of 6 doses of omalizumab were included in the analysis. Flow chart of study patients is depicted in Figure 1.

### 3.1. Demographic and baseline characteristics

Among the study population, 46 (56.8%) were female, with an average age of 32.28 years (SD, ±8.253). Forty (49.4%) patients experienced wheals only, 30 (37.03%) exhibited dermographism alongside wheals, and 11 (13.5%) had wheals accompanied by angioedema. The median duration of illness was 24 months (IQR: 7.5–33 months). Baseline clinicodemographic parameters of the study population are detailed in Table 1.

**Table 1.** Demographic characteristics of the patients across various response groups

Baseline characteristics	Total, N = 81	Early responders, n = 53 (65.4)	Late responders, n = 17 (21.0)	Responders with frequent dosing, n = 6 (7.4)	Nonresponders, n = 5 (6.2)
Age, mean (SD), y	32.28 (8.253)	32.17 (7.924)	30.76 (7.2)	33.83 (11.4)	36.8 (11.6)
Female, n (%)	46 (56.8)	29 (41.4)	10 (58.8)	4 (66.7)	4 (80)
Male, n (%)	35 (43.2)	24(34.3)	7 (41.2)	2 (33.3)	1 (20)
Disease characteristics, n (%)					
Wheals	40 (49.4)	31 (58.5)	2 (11.8)	5 (83.3)	3 (60)
Wheals and dermographism	30 (37.1)	18 (33.9)	9 (52.9)	0	2 (40)
Wheals and angioedema	11 (13.5)	4 (7.54)	6 (35.3)	1 (16.7)	0
Time from onset of disease to presentation, median (range), mo	24 (7.5–33)	12 (9–18)	15 (9–21)	8.5 (6–12)	36 (30–54)
Treatment before omalizumab, n (%)					
Antihistamines only					
Levocetirizine	35 (43.2)	27 (50.9)	5 (29.4)	3 (50)	0
Bilastine	15 (18.5)	10 (18.9)	4 (23.5)	1 (16.7)	0
Fexofenadine	11 (13.6)	8 (15.1)	2 (11.8)	1 (16.7)	0
Oral steroids	9 (11.1)	4 (7.5)	4 (23.5)	0	1 (20)
Phototherapy	2 (2.5)	2 (3.8)	0	0	0
Cyclosporine	7 (8.6)	1 (1.9)	2 (11.8)	1 (16.7)	3 (60)
Azathioprine	2 (2.5)	1 (1.9)	0	0	1 (20)
Median number of omalizumab doses received	6 (6–8)	6 (6–8)	6.0 (6–7)	6.0 (6–8)	9 (8–12)

SD, standard deviation.

Seventy-two patients (69.1%) had elevated serum IgE levels (>100 IU/mL), with 14 (17.3%) surpassing (>500 IU/mL), 2 (2.5%) exceeding (>1,000 IU/mL), and 9 (11.1%) presenting with low IgE levels (<100 IU/mL). Nine of fifty-eight (11.1%) patients demonstrated increased CRP levels, while 17 of 50 (21.0%) showed decreased serum vitamin D levels within the study cohort. Four of forty-nine (4.9%) patients had elevated d-dimer levels, 14 of 56 (17.3%) tested positive for anti-TPO, and 16 of 54 (19.8%) showed positive ANA results.

The median serum IgE levels were 270 IU/mL (IQR: 145–471), the median CRP was 2.0 (IQR: 1–4), and the mean vitamin D level was 23.56 (SD, ±8.992). The median d-dimer level was 60 (IQR: 25–143), and the median baseline UAS7 was 32 (IQR: 21–42). UAS7 after the first dose was 16 (IQR: 7–30), and after 6 doses, it reduced to 0 (IQR: 0–6). The median number of omalizumab doses received was 6 (range: 6–8).

Before treatment with omalizumab, all patients received AH_1_. Thirty-five (43.2%) received levocetirizine only, 15 (18.5%) were prescribed bilastine only, and 11 (13.6%) received fexofenadine only, while 9 patients (11.1%) required steroids along with AH_1_. Additionally, 2 (2.5%) patients underwent phototherapy, while 7 (8.6%) were treated with cyclosporine and 2 (2.5%) patients were given azathioprine along with AH_1_.

### 3.2. Response to omalizumab

Following the initial dose of omalizumab, 53 (65.4%) patients attained a UAS7 score of <6. Subsequently, 17 (21.0%) patients reached a UAS7 score of <6 between the second and third doses. For those patients who did not achieve a UAS7 score of <6 after the third dose, the injection frequency was escalated to every 3 weeks. Six (7.4%) patients achieved remission with the increased dosing frequency. However, 5 (6.2%) patients continued to exhibit persistently high UAS7 scores (>6) even after the dosage frequency was escalated.

**Figure 2.**
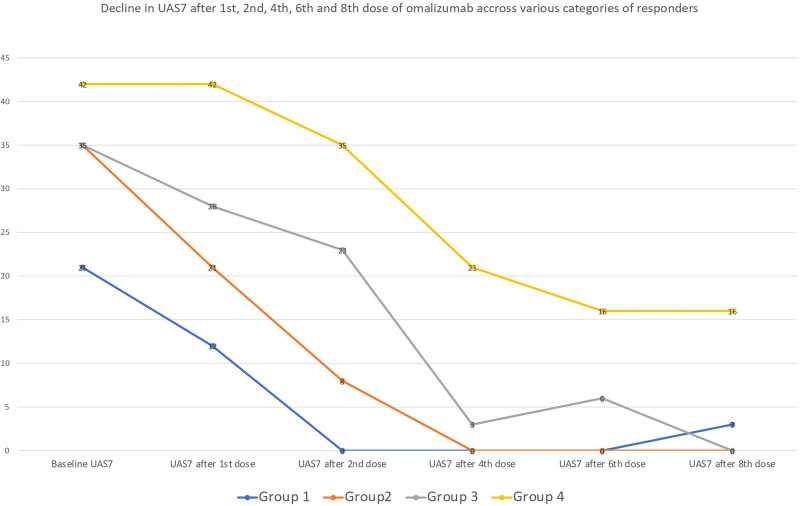
Graph showing median UAS7 at baseline, first, second, fourth, and sixth dose of omalizumab among different groups. UAS7, 7-day urticaria activity score.

Patients were then categorized based on their response to omalizumab, resulting in 53 individuals classified as early responders (group 1), 17 as late responders (group 2), 6 requiring 3-weekly dosing for disease control (group 3), and 5 nonresponders (group 4). Figure 2 illustrates the trends in median UAS7 score among 4 groups during the first 6 months of omalizumab therapy.

### 3.3. Response categories and correlation with baseline characteristics

Kruskal–Wallis test and chi-square test were performed to assess the relationship between baseline parameters and 4 response groups. The distribution of serum IgE (*P* < 0.001), d-dimer (*P* = 0.001), anti-TPO (*P* = 0.031), ANA (*P* = 0.047), and baseline UAS7 (*P* = 0.013) was significantly different across 4 response groups, whereas there was no statistically significant difference among the groups in terms of age (*P* = 0.639), CRP (*P* = 0.184), and serum vitamin D levels (*P* = 0.447). Figure 2 illustrates the median baseline laboratory parameters among the 4 response groups.

The proportion of patients exhibiting elevated IgE levels was notably higher in group 1 compared with the other 3 groups. This contrast was statistically significant when group 1 compared with group 2 (*P* = 0.001), group 3 (*P* = 0.004), and group 4 (*P* < 0.001), after Bonferroni correction for multiple tests. However, the disparity in IgE levels was not statistically significant among groups 2, 3, and 4.

The proportion of patients with elevated d-dimer was higher in groups 3 and 4, and this difference across the categories was statistically significant (*P* = 0.001). Pairwise analysis showed statistically significant differences between groups 2 and 4 (*P* = 0.12) and groups 2 and 3 (*P* = 0.01). Regarding baseline UAS7 scores, there was a significant difference observed across different categories (*P* = 0.013). Pairwise analysis indicated a statistically significant difference between groups 1 and 3 (*P* = 0.034).

**Figure 3.**
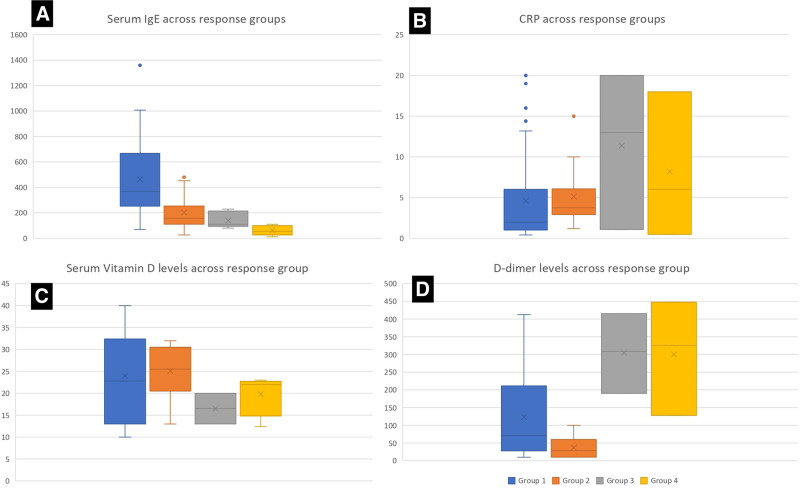
Box and whisker plot showing the distribution of baseline characteristics across different response groups.

Overall, factors associated with failure to attain response with a single dose of omalizumab (early response) included the presence of ANA (*P* = 0.04), anti-TPO antibody (*P* = 0.031) positivity, and prior treatment with immunosuppressants before omalizumab (*P* = 0.006). However, no statistically significant correlations were found between gender (*P* = 0.944) and poor treatment response after the first dose of omalizumab. Further details are outlined in Table 2 and Figure 3.

**Table 2.** Characteristics of patients stratified based on response to omalizumab

Lab parameters	Total	Group 1	Group 2	Group 3	Group 4	*P* value
Age, mean (SD), y	32.28 (8.253)	32.17 (7.924)	30.76 (7.2)	33.83 (11.4)	36.8 (11.6)	0.639*
Gender						0.701†
Female, n (%)	46 (56.8)	29 (41.4)	10 (58.8)	4 (66.7)	4 (80)	
Male, n (%)	35 (43.2)	24(34.3)	7 (41.2)	2 (33.3)	1 (20)	
Serum IgE (n = 81)						**<0.001** *
<100	9 (11.1)	2 (3.8)	2 (11.8)	1 (16.7)	4 (80)	
100–500	56 (69.1)	35 (66.0)	15 (88.2)	5 (83.3)	1 (20)	
500–1,000	14 (17.3)	14 (26.4)	0	0	0	
>1,000	2 (2.5)	2 (3.8)	0	0	0	
CRP (n = 57)						0.184*
Normal	39 (68.4)	28 (71.8)	8 (66.7)	1 (33.3)	2 (66.7)	
High	18 (31.6)	11(28.2)	4 (33.3)	2 (66.7)	1 (33.3)	
Serum vitamin D (n = 50)						0.447*
Normal	33 (66)	21 (61.8)	8 (80)	1 (50)	3 (75)	
Low	17 (34)	13 (38.2)	2 (20)	1 (50)	1 (25)	
d-Dimer (n = 49)						**0.001** *
Normal	40 (81.6)	26 (81.3)	11 (100)	1 (33.3)	2 (66.7)	
High	9 (18.4)	6 (18.8)	0	2(66.7)	1 (33.3)	
Anti-TPO (n = 56)						**0.031** †
Normal	42 (75)	31 (86.1)	8 (66.7)	2 (40)	1 (33.3)	
Elevated	14 (25)	5 (13.9)	4 (33.3)	3 (60)	2 (66.7)	
ANA (n = 54)						**0.047** †
Normal	38 (70.4)	29 (80.6)	7 (63.6)	1 (25)	1 (33.3)	
Elevated	16 (29.6)	7 (10.0)	4 (36.4)	3 (75)	2 (66.7)	
Median baseline UAS7 (IQR)	32 (21–42)	21 (21–35)	35 (32–42)	35 (21–42)	42 (35–42)	**0.013** *

ANA, antinuclear antibodies; CRP, C-reactive protein; IQR, interquartile range; SD, standard deviation; TPO, thyroid peroxidase; UAS7, 7-day urticaria activity score. A statistically significant difference was observed in serum IgE, D-Dimer, Anti-TPO, ANA, and median UAS7 across the four different response groups (highlighted in bold).

* Kruskal–Wallis test.

† Chi-square test.

### 3.4. Recurrence after initial treatment response

A total of 22 patients (27.1%) had exacerbation during the course of the treatment. Among these 14 patients (63.6%) necessitated a short course of oral steroids to manage exacerbations, whereas 6 patients (27.2%) required a mere change in AH_1_ (levocetirizine to bilastine). Additionally, 2 patients (2.09%) necessitated supplementary immunosuppressants like low-dose cyclosporine (3 mg/kg) to control disease activity. Nonsteroidal anti-inflammatory drugs intake was identified as a common triggering factor for recurrence in 2 patients, no exacerbating factors were apparent among other cases.

### 3.5. Adverse effects of omalizumab

Most common adverse effect was local pain at the injection site seen in 7 (8.64%) patients. No major adverse effects were noted in the omalizumab treatment group.

## 4. Discussion

CSU presents as a persistent and distressing condition, causing physical discomfort and disrupting daily life with its unpredictable flare-ups. Recent research has highlighted the existence of distinct CSU endotypes—autoallergic and autoimmune—which may differ in treatment response [11]. Omalizumab, endorsed in current guidelines for managing AH1-resistant CSU, alongside AH_1_, has demonstrated symptom control in 87.0% to 91.4% of CSU cases in real-world settings [12]. Despite this, certain factors, such as low baseline serum IgE levels, have been linked to poor response to omalizumab, reflecting the complexity of treatment outcomes [13]. This real-world study from Northern India sheds light on omalizumab’s efficacy and explores correlations between patient characteristics and treatment response patterns, contributing to our understanding of its clinical use.

In our study population, a notable 65% of patients experienced good response (UAS7 <6), following just a single dose of omalizumab (group 1). These responders exhibited distinct characteristics, including elevated median serum IgE levels and low d-dimer levels, suggesting an autoallergic type of urticaria. Remarkably, the proportion of patients with positive anti-TPO and ANA antibodies was significantly lower in early responders compared with groups 3 and 4. Elevated serum IgE levels, a well-established indicator of favorable response to omalizumab, were prominently observed in this group, aligning with previous research findings [11]. A meta-analysis revealed that both complete and partial responders had notably elevated serum IgE levels compared with nonresponders, with no significant variance observed between complete and partial responders [14]. In contrast, Ghazanfar et al. [15] reported that various patient-specific factors were not significantly associated with omalizumab response at 3 months, indicating the complexity of treatment responsiveness in CSU.

A subset of patients, representing approximately one-fifth of the cohort, demonstrated a delayed response between 4 and 12 weeks following the administration of the second or third doses of omalizumab (group 2). This subgroup exhibited mildly elevated serum IgE levels, typically ranging between 100 and 500 IU/L, and notably, had a higher incidence of concurrent chronic inducible urticaria (CIndU) alongside CSU. Previous research has indicated that while omalizumab tends to be effective in CIndU patients, the response rates are generally lower compared with CSU [16]. Furthermore, patients with CIndU are less likely to respond early to omalizumab compared with those with CSU [17]. These findings collectively suggest that the presence of concomitant CIndU may contribute to delayed treatment responses to omalizumab among patients with CSU.

While the approved dosage for omalizumab is 300 mg every 4 weeks, some studies indicate the need for increased dosing to 450/600 mg on a similar schedule [18]. The financial burden, particularly in the context of India, poses a significant challenge. In such circumstances, an alternative approach could involve more frequent injections, with a 3-week interval between doses.

Six patients in our cohort benefited from a reduction of dosage interval to 3 weeks (group 3), showcasing mildly elevated serum IgE levels alongside elevated CRP, low vitamin D, elevated d-dimer, and positive anti-TPO and ANA, reminiscent of coexistent autoimmune and autoallergic endotype of urticaria. Similar observations have been reported by Asero et al. [19], who correlated late response to omalizumab with the coexistence of both IgE and IgG autoantibodies in CSU patients. In CSU patients, a rapid response to omalizumab has been linked to higher levels of total IgE and a slow or absent response has been associated with IgG autoimmunity [20]. Omalizumab’s ability to bind circulating IgE and detach them from the receptor, resulting in downregulation of the receptor, may contribute to a delayed response, particularly in patients with high IgG autoantibodies [21]. The coexistence of IgE and IgG autoantibodies may lead to unpredictable responses to the drug, possibly depending on the balance between these autoimmune responses or the specific clinical effects of the autoantibodies. Other patient-related factors suggesting a potential benefit from updosing, as indicated in previous literature, include higher BMI and lower preomalizumab UCT scores [22].

In our study cohort, 5 patients did not respond even with frequent dosing, displaying low serum IgE levels, elevated d-dimer, positive ANA, and anti-TPO. This subset of patients did not respond to frequent dosing of omalizumab unlike group 3. These patients had characteristics indicative of autoimmune urticaria. Several prior studies have demonstrated poor response to omalizumab in patients with autoimmune endotype of CSU [23]. The results of the Profiling Urticaria for the identification of Subtypes study showed that less than 10% of patients with CSU have autoimmune CSU and that these patients tend to have more severe disease, low levels of total IgE, and high levels of anti-TPO autoantibodies [24]. Further studies pointed toward IgM and/or IgA autoantibodies against FcεRI [25], eosinopenia [26], low levels of total IgA [27], and poor and/or slow response to conventional treatment with AH_1_ and omalizumab as markers of autoimmune CSU [28].

Our study demonstrates the remarkable efficacy of omalizumab in treating CSU in real-world settings, with an impressive 93.8% of patients exhibiting a positive response within 6 months of initiating therapy. Moreover, the incidence of adverse effects was minimal, underscoring the safety profile of omalizumab. However, the high cost of treatment remains a significant barrier to accessibility, particularly in the Indian context. Despite this limitation, our findings underscore the transformative impact of omalizumab on the management of urticaria. Importantly, our study highlights the potential utility of routinely assessing biomarkers such as serum IgE and anti-TPO levels in CSU patients, offering valuable insights for prognostication and treatment decision-making. Moving forward, further research into biomarker-based endotypes of urticaria holds promise for the development of personalized treatment approaches tailored to the specific disease characteristics of individual patients.

### 4.1. Strengths and limitations

Strengths include adherence to standardized protocols, detailed assessment of treatment response, and analysis of potential biomarkers such as serum IgE levels. However, limitations include the retrospective design, lack of a control group, and potential biases in data collection, limiting generalizability. Despite these limitations, the study contributes valuable real-world evidence to the understanding of omalizumab therapy in CSU and highlights the need for future prospective studies to address these limitations and further validate findings.

## 5. Conclusion

Our study highlights the effectiveness of omalizumab in treating CSU in Northern India, with a high response rate observed within 6 months of therapy initiation. While omalizumab demonstrated significant efficacy and minimal adverse effects, cost remains a barrier to accessibility. Routine assessment of biomarkers such as serum IgE and anti-TPO levels in CSU patients could aid in prognostication. Prospective studies are needed to validate these findings and further advance CSU management.

## Conflicts of interest

The authors have no financial conflicts of interest.

Thammannagowda Prarthana: Data acquisition, data analysis and interpretation, manuscript drafting and editing.

Hitaishi Mehta: Data acquisition, data analysis and interpretation, manuscript editing.

Anuradha Bishnoi: Study design and manuscript editing.

Davinder Parsad: Conception and manuscript editing.

Muthu Sendhil Kumaran: Conception and design, manuscript editing and approval.
